# Immunologic Corneal Graft Rejection after Administration of Topical Latanoprost: a Report of Two Patients

**Published:** 2011-04

**Authors:** Kouros Nouri-Mahdavi, Mohammad-Ali Javadi, Mohammad-Reza Jafarinasab

**Affiliations:** 1 Jules Stein Eye Institute, University of California Los Angeles, Los Angeles, California, USA; 2 Ophthalmic Research Center, Shahid Beheshti University of Medical Sciences, Tehran, Iran

**Keywords:** Corneal Graft Rejection, Latanoprost, Penetrating Keratoplasty

## Abstract

**Purpose:**

To report endothelial corneal graft rejection after administration of topical latanoprost eye drops.

**Case Report:**

Two eyes of two patients with a history of multiple intraocular procedures prior to penetrating keratoplasty developed endothelial graft rejection one month after administration of topical latanoprost. Cystoid macular edema developed simultaneously in one patient.

**Conclusion:**

Latanoprost may trigger endothelial graft rejection in susceptible eyes.

## INTRODUCTION

Latanoprost (Xalatan, Pharmacia and Upjohn, Kalamazoo, MI, USA) has been associated with a number of ocular side effects including increased iris pigmentation, elongation of cilia, hypertrichosis, and skin pigmentation.^1^,^2^ In addition, topical latanoprost has been reported to cause uveitis, cystoid macular edema (CME), and recurrence of herpes simplex keratitis.^3^–^7^ Cystoid macular edema has mostly occurred after complicated cataract surgery. Herein, we report the occurrence of endothelial corneal graft rejection shortly after starting topical latanoprost in 2 eyes with prior penetrating keratoplasty (PKP).

## CASE REPORTS

### Patient 1

**Figure 1:**
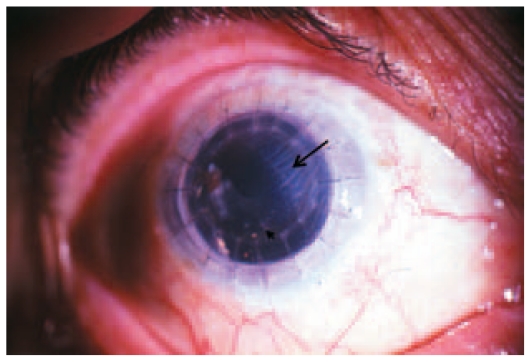
Immunologic graft rejection one month after starting latanoprost in case 1. The larger arrow shows the border between the clear and edematous areas of the graft. The small arrow points to inferior keratic precipitates.

A 68-year-old woman underwent PKP a few months after complicated cataract surgery in the right eye in 1995. She had a second PKP in the same eye in June 2000. After intraocular pressure (IOP) became uncontrolled, latanoprost was added to her prescription of timolol. Two weeks later, best-corrected visual acuity (BCVA) was 20/400, slit-lamp examination revealed a clear, avascular graft and a quiet eye, IOP was 57 mmHg. A polymethyl methacrylate (PMMA) intraocular lens was observed, centered over an open posterior capsule. Vitreous incarceration in the cataract incision was present superonasally along with extensive iridocorneal adhesions. The optic disc seemed mildly saucerized. Moderate pigmentary changes were evident in the macula. After adding acetazolamide, IOP was reduced to 16 mmHg. Topical dorzolamide was substituted for acetazolamide with maintenance of IOP control. Two weeks later, the patient returned with a decrease in BCVA to counting fingers at 1 m having mistakenly used latanoprost four times daily. Tonometry revealed IOP of 10 mmHg. Sectoral corneal edema, multiple white keratic precipitates, and mild anterior chamber reaction were observed (Figure 1). Cystoid macular edema was suspected after fundus examination. Latanoprost was immediately discontinued and graft rejection was treated with corticosteroids. Fluorescein angiography was performed the next day and confirmed the presence of CME. The rejection episode gradually improved over the next few weeks together with resolution of corneal edema, inflammatory signs and CME while BCVA improved to 20/400.

### Patient 2

A 45-year-old man with a history of bilateral trabeculectomy for primary open-angle glaucoma, underwent repeat trabeculectomy in his right eye with mitomycin C by one of the authors (MAJ). One year later, a third trabeculectomy with adjunctive mitomycin C was performed on the same eye because of uncontrolled IOP. Postoperatively, the cornea decompensated gradually and a PKP was performed. IOP increased gradually after PKP and timolol and dorzolamide were started. At that point, the graft was clear and avascular. Three months after PKP, latanoprost was started because of uncontrolled IOP. One month later, epithelial and endothelial rejection occurred in the same eye. The rejection episode partially responded to topical steroids and cyclosporine. Moderate residual corneal edema and visual acuity of counting fingers prompted a second graft in this eye 11 months after the rejection episode. Five months after the second PKP, the graft remained clear and IOP was under control on timolol and dorzolamide.

## DISCUSSION

We observed the occurrence of corneal graft rejection shortly after initiating topical latanoprost in 2 eyes with previous PKP. To our knowledge, this has not been previously reported in the literature.

Immunologic graft rejection is particularly common during the first year after PKP^8^ and both patients could be considered at high risk for graft rejection. Patient 1 had extensive peripheral anterior synechiae and vitreous incarceration into the cataract incision, while the second patient had multiple prior procedures. However, both patients had avascular corneas before PKP. In addition, more than one year had elapsed after PKP in patient 1 when graft rejection suddenly occurred. The temporal sequence of graft rejection immediately after starting latanoprost, is strongly suggestive of a possible relationship.

Since the early days of development of prostaglandins for treatment of glaucoma, the pro-inflammatory effects of such compounds have been a concern. However, a number of early pilot studies^9^–^11^ suggested that latanoprost has little, if any, pro-inflammatory effect. The highly selective affinity of latanoprost for FP receptors, the subtype of receptors most sensitive to prostaglandin F, makes vascular effects for latanoprost unlikely, even at high concentrations.^12^ With widespread use of latanoprost, it was shown to be rarely associated with side effects such as CME and attacks of iritis or anterior uveitis.^3^–^6^ However, latanoprost has been shown to lead to disruption of the blood aqueous-barrier and an increased incidence of angiographic CME in the early postoperative period.^13^ In a clinical trial comparing latanoprost, travoprost, and bimatoprost to placebo in aphakic and pseudophakic eyes, eyes receiving one of the prostaglandins had higher flare values and a higher incidence of CME.^14^ Both complications usually occur in eyes with risk factors such as previous complicated surgery with a disrupted or absent posterior capsule, anterior chamber IOL, or history of anterior uveitis. The common denominator for all of the above risk factors is persistent disruption of the blood-aqueous barrier. However, Schumer and colleagues^15^ have raised doubts whether the reports in the literature actually represent side effects of latanoprost. Our first patient had undergone complicated cataract surgery with disruption of the posterior capsule and vitreous incarceration into the wound, along with two PKPs while the second patient had multiple trabeculectomies prior to PKP. We hypothesize that multiple, complicated interventions probably led to chronic breakdown of the blood-aqueous barrier in these patients. Although a direct cause-and-effect relationship cannot be proven with only two patients, the short time interval from initiation of latanoprost to graft rejection (about a month in both patients) may suggest an association. The first patient had erroneously used a four-fold higher than usual dose of latanoprost for at least two weeks, which might have potentiated any pro-inflammatory effect of latanoprost. Interestingly, this patient developed CME at about the same time as graft rejection, adding further support to an association between latanoprost and immunologic graft rejection. It may be speculated that topical administration of latanoprost and breakdown of the blood-aqueous barrier could have acted synergistically and led to graft rejection in these eyes.

We suggest that topical latanoprost may trigger immunologic graft rejection in susceptible eyes. Until further studies are performed, it seems prudent to use topical latanoprost with caution in glaucomatous eyes after PKP, especially in the presence of chronic breakdown of the blood-aqueous barrier and in eyes with other risk factors for graft rejection.
